# Serum Markers of Oxidative Stress and Antioxidant Status in Keratoconus: Ischemia-Modified Albumin, Malondialdehyde, and Total Thiol Levels

**DOI:** 10.3390/medicina62040682

**Published:** 2026-04-02

**Authors:** Serek Tekin, Erbil Seven, Canan Demir, Halit Demir, Muhammed Batur, Muhammet Derda Özer, Tekin Yaşar

**Affiliations:** 1 Department of Ophthalmology, Faculty of Medicine, Yuzuncu Yil University, 65080 Van, Turkey; erbilseven@gmail.com (E.S.); muhammetderda@gmail.com (M.D.Ö.); 2 Vocational School of Healthcare, Yuzuncu Yil University, 65080 Van, Turkey; canandemir@yyu.edu.tr; 3 Department of Biochemistry, Faculty of Science, Yuzuncu Yil University, 65080 Van, Turkey; halitdemi@gmail.com; 4 Urartu Eye Medical Center, 65100 Van, Turkey; muhammedbatur@gmail.com; 5 Department of Ophthalmology, Beyoglu Eye and Research Hospital, 34421 Istanbul, Turkey; tekinyasar@yahoo.com

**Keywords:** keratoconus, oxidative stress, ischemia-modified albumin, malondialdehyde, total thiol

## Abstract

*Background and Objectives:* Keratoconus (KC) is a progressive corneal ectatic disorder characterized by stromal thinning, irregular astigmatism, and visual impairment. Increasing evidence suggests that oxidative stress plays a significant role in the pathogenesis of KC. Biomarkers such as ischemia-modified albumin (IMA), malondialdehyde (MDA) and total thiol (TT) have been widely used to assess oxidative status. This study aims to evaluate the role of oxidative stress in KC disease by comparing serum levels of IMA, MDA, and TT between KC patients and healthy controls. *Material and Methods:* Forty patients diagnosed with KC and 43 healthy individuals of similar age and gender were included in the study. Patients with KC were classified in 4 stages according to the modified Krumeich KC classification system. IMA, MDA, and TT levels were compared in serum samples from patient and control groups. *Results:* Serum IMA levels were 2.52 ± 0.07 in the KC group and 1.2 ± 0.03 in the control group. The difference was statistically significant (*p* < 0.001). While serum MDA levels were 1.68 ± 0.05 in the KC group, they were 0.74 ± 0.04 in the control group. The difference was statistically significant (*p* < 0.001). Serum TT levels were 0.82 ± 0.41 in the KC group and 2.23 ± 0.04 in the control group. The difference was statistically significant (*p* < 0.001). *Conclusions:* Elevated serum IMA and MDA levels, together with decreased TT levels, in patients with KC are likely associated with increased oxidative stress. These parameters may serve as auxiliary biomarkers for evaluating disease pathophysiology and may represent potential targets for future diagnostic and therapeutic strategies.

## 1. Introduction

Keratoconus (KC) is a progressive corneal disorder characterized by corneal thinning, which leads to irregular astigmatism and decreased visual acuity. Thinning generally occurs in the central and inferior cornea [1]. The disease typically presents in early childhood and may progress into the 4th decade of life [2]. While its prevalence is 265 in 100,000, its incidence is 1:7500 per year, according to epidemiological studies [3]. The etiology of the disease is not clearly known. Genetic and environmental factors are thought to act together in its etiology. It is reported that 6–10% of cases exhibit autosomal-dominant inheritance [4].

Environmental factors implicated in KC etiology include corneal exposure to ultraviolet (UV) radiation, ocular rubbing, and allergic reactions [1]. Continuous exposure of the cornea to ultraviolet radiation leads to oxidative stress and the excessive production of reactive oxygen species (ROS) [5]. The resulting ROS can interact with cellular membranes, DNA, and proteins, disrupting cellular function. Numerous studies in the literature report oxidative damage in the eyes with KC [5,6]. Oxidative stress leads to tissue degeneration, which may result in stromal thinning and loss of the Bowman’s layer in the cornea with KC [4,6]. In healthy corneas, there are antioxidant enzymes that destroy the formed ROS without damaging the corneal cells. These are enzymes such as superoxide dismutase (SOD), catalase (CAT), glutathione peroxidase (GPx), and glutathione reductase (GR). In healthy corneal tissue, there is a balance between ROS formed and ROS eliminated by antioxidants, but this balance is disrupted in the corneas with KC [5,6,7,8]. There are many biochemical markers used to assess oxidative stress. Biomarkers such as ischemia-modified albumin (IMA), malondialdehyde (MDA), and total thiol (TT) are frequently used to determine the extent of oxidative stress.

Ischemia, hypoxia, acidosis, free radicals, and ROS cause damage to the N-terminal of albumin protein, and albumin fails to bind bivalent metals [9]. Human serum albumin with reduced binding capacity resulting from these events is called ischemia-modified albumin (IMA) [10]. In recent years, IMA has been used as a biomarker of ischemic events and oxidative stress [11,12].

ROS, which result from oxidative stress, stimulate lipid peroxidation in corneal cells. Reactive aldehydes such as malondialdehyde (MDA) and 4-hydroxy-2-nonenal (HNE) appear after lipid peroxidation. Therefore, MDA is a marker of oxidative stress [13].

TT levels are an important component of systemic antioxidant defense and reflect the organism’s redox status. Total thiols function as essential antioxidants by directly scavenging reactive oxygen species and by maintaining cellular redox balance through the reversible oxidation–reduction of sulfhydryl (–SH) groups. They also protect proteins and enzymes from oxidative damage and contribute to redox signaling by forming and reducing disulfide bonds. Through these mechanisms, the total thiol pool plays a central role in cellular defense against oxidative stress [14].

This study aims to evaluate the relationship between KC disease and serum IMA, MDA, and TT levels. Numerous studies have assessed the role of oxidative stress in KC pathogenesis and its relationship with oxidative stress biomarkers [4,5,8]. However, we have not found a study that evaluates the relationship between KC and IMA as oxidative stress markers. To the best of our knowledge, this is the first study to evaluate IMA in KC in conjunction with both lipid peroxidation (MDA) and thiol-based antioxidant capacity (TT), thereby providing a more integrated assessment of systemic oxidative balance in KC.

## 2. Materials and Methods

### 2.1. Ethical Considerations

This study was conducted at the Department of Ophthalmology, Van Yuzuncu Yil University. Approval was obtained from the Van Yuzuncu Yil University Clinical Research Ethics Committee (21 May 2020—Number: 02). In the methods used, the principles of the Declaration of Helsinki were adhered to. All participants in the study were informed, and both verbal and written informed consent were obtained.

### 2.2. Study Population

Forty individuals who were identified by random sampling among the patients followed up for KC in our clinic were included as the patient group, and 43 healthy individuals who were similar to the patient group in terms of age and gender and who did not have any eye disease other than refractive error were included as the control group in the study.

In both groups, those with diseases such as diabetes mellitus (DM), hypertension, cardiovascular disease, hyperlipidemia, rheumatic diseases, malignancy, bronchial asthma, smoking and/or consuming alcohol, using steroids, chemotherapeutic, diuretic, anti-inflammatory, antioxidant drugs or vitamin supplements were excluded from the study, as well as those diagnosed with an eye disease other than KC on eye examination or those with previous surgical history related to the eye. In the control group, individuals with spherical and/or cylindrical refractive errors exceeding ±1.5 D, and those with KC or suspected KC on clinical examination and corneal topography, were excluded from the study.

Patients with KC were classified in 4 stages according to the modified Krumeich KC classification system [15]. In patients with KC in both eyes, the more advanced eye was included in the classification.

The best-corrected visual acuity of all participants was obtained (Snellen chart). A complete ophthalmological examination was performed, including examination by biomicroscope, intraocular pressure measurement, and dilated fundus examination. Corneal topography was taken (Orbscan Hz; Technolas, München, Germany).

### 2.3. Sample Collection

A total of 3 mL of venous blood was collected from all participants, centrifuged at 5000 rpm for 10 min, and the serum was separated. Serum samples were stored at −80 °C until analysis. Then, IMA, MDA, and TT levels were determined in these sera.

### 2.4. IMA Assay

Serum IMA levels were determined using the method described by Bar-Or et al. [10], which measures the albumin-bound cobalt level in participants’ serum samples. To measure IMA, 50 µL of cobalt chloride was added to 200 µL of serum; the mixture was gently shaken and incubated for 10 min to ensure proper binding of cobalt to albumin. Then, 50 µL of 1.5 mg/mL dithiothreitol (DTT) (Sigma-Aldrich, München, Germany) was added as the coloring reagent, and the binding reaction was stopped by adding 1.0 mL of 0.9% NaCl after 2 min. A colorimetric control was prepared for each sample. 50 µL distilled water was used instead of 50 µL 1.5 mg/mL DTT for control samples. The resulting color complex was measured spectrophotometrically at 470 nm. The results were reported as absorbance units (Abs.U).

### 2.5. MDA Assay

MDA, a peroxidation product formed by the reaction of fatty acids with free radicals, was measured as a result of turning into a colored form with thiobarbituric acid (TBA) [16]. A total of 200 µL of serum was collected into a single tube. Then, 800 µL phosphate buffer, 25 µL butylhydroxytoluene (BHT) solution, and 500 µL of 30% trichloroacetic acid (TCA) were added. The tubes were vortex-mixed and kept on ice for 2 h. It was then centrifuged at 2000 rpm for 15 min. A total of 1 mL of the supernatant was collected and transferred to separate tubes. Then, 75 µL EDTA and 25 µL TBA were added to these. The tubes were mixed in a Vortex and held in a hot water bath for 15 min. Then, it was brought to room temperature, and absorbance was measured at 532 nm in a UV–Vis spectrophotometer.

### 2.6. TT Assay

This method is based on the reaction of Ellman’s reagent (DTNB) with sulfhydryl groups, which yields 2-nitro-5-mercaptobenzoic acid via reduction. The anionic form of this compound exhibits a pale yellow color, enabling spectrophotometric detection of thiol groups in plasma. Reduced glutathione served as the calibration standard [17].

Blood samples collected in EDTA tubes were first centrifuged at 3500 rpm for 5 min to obtain plasma. A 0.5 mL volume of plasma was then transferred into centrifuge tubes. Afterward, 1.5 mL of Tris buffer (0.2 M, pH 8.2) was added, followed by 0.1 mL of a 0.01 M DTNB solution. The mixture was thoroughly homogenized using a vortex mixer. Subsequently, 7.9 mL of methanol was added, and the tubes were allowed to stand for 15 min to develop color.

The samples were then centrifuged again at 4500 rpm for 10 min at room temperature. Absorbance was measured at 412 nm using a blank prepared by replacing plasma with disodium EDTA and replacing the DTNB solution with distilled water.

### 2.7. Statistical Analysis

Statistical Package for the Social Sciences (SPSS) version 23.0 was used for statistical analysis. Descriptive statistics were used to calculate the mean and standard deviation for age, gender, IMA, MDA, and TT levels in the patient and control groups. The Shapiro–Wilk test was used to assess whether the data were normally distributed. The Mann–Whitney U test was used to compare serum IMA, MDA, and TT levels in the KC and control groups. Spearman’s rank correlation was performed to assess the association among serum IMA, MDA, and TT values in patients with KC. Multinomial logistic regression analysis was performed to compare serum IMA, MDA, and TT levels according to KC stages. A *p*-value below 0.05 was considered statistically significant.

## 3. Results

Of 40 patients with KC included in the study, 26 were female (65%) and 14 were male (35%). Of 43 healthy individuals in the control group, 24 were female (55.8%) and 19 were male (44.2%). While the mean age of the KC group was 22.6 ± 4.9 years (18–35), that of the control group was 22.8 ± 2.7 years (18–27). There were no statistically significant differences between the two groups in gender or age (*p* = 0.39 and *p* = 0.17, respectively). Among patients with KC, 11 were in stage 1, 21 in stage 2, 6 in stage 3, and 2 in stage 4.

Mean serum IMA and MDA levels were significantly higher, whereas mean serum TT levels were significantly lower in the KC group compared to controls (*p* ˂ 0.001, *p* ˂ 0.001, *p* ˂ 0.001, respectively). The mean serum IMA, MDA, and TT values in the KC and control groups are summarized in Table 1.

Spearman’s rank correlation was performed to assess the association among IMA, MDA, and TT. No statistically significant relationship was found between them (*p* = 0.09, r = 0.21).

Multinomial logistic regression analysis was performed to evaluate the relationship between KC stage and IMA, MDA, and TT values. No statistically significant association was found between disease stage and IMA, MDA, or TT (*p* > 0.05).

To address the limited number of advanced-stage cases, stages 3 and 4 patients were combined into a severe KC group. However, no significant differences were observed among stage 1, stage 2, and severe KC groups, which may still reflect limited statistical power or suggest that oxidative stress markers are more closely associated with disease presence rather than severity.

## 4. Discussion

In this study, the serum levels of oxidative stress markers IMA and MDA, and the antioxidant indicator TT, were compared between KC patients and healthy controls. The serum IMA and MDA levels were higher, while the serum TT level was lower in the KC group.

KC is not a disease with a clearly defined etiology. Oxidative stress, increasingly considered an environmental factor, is thought to induce the disease by damaging corneal cells [5,18]. This view is supported by the observation that ROS, a product of oxidative stress, is significantly higher in corneas from patients with KC than in normal corneas [4,6]. Similarly, in some blood studies, serum levels of oxidative stress biomarkers were elevated in patients with KC [8,19].

IMA is a biomarker formed by the modification of albumin as a result of ROS interactions. ROS are generated by oxidative stress, hypoxia, and acidosis, and by the presence of free radicals and free iron [9,10]. IMA is used as a marker in ischemic events such as myocardial ischemia and acute coronary syndrome [20]. However, recent studies have reported high IMA levels in diseases other than ischemic heart disease, such as DM, hyperlipidemia, chronic kidney disease, obesity, and systemic sclerosis [21,22,23,24]. This indicates that high IMA levels are not organ- or tissue-specific but may serve as a general indicator of oxidative stress [24].

Studies have investigated the relationship between IMA and certain eye diseases. Most of these studies concern diabetic retinopathy. However, to our knowledge, our study is the first to demonstrate the relationship between IMA and KC. Gulpamuk et al. [25] divided 122 patients with DM into 3 groups according to their retinal involvement and found that IMA levels were significantly higher in the group with proliferative diabetic retinopathy compared to the non-proliferative group and the group without retinopathy. The reason for this was attributed to ischemia and oxidative stress. Kirboga et al. [26] compared the serum IMA levels in 22 patients with non-proliferative diabetic retinopathy and in the control group, and found that IMA levels were higher in the group with retinopathy. In a similar study, Turk et al. [27] found that IMA levels were significantly higher in patients with diabetic retinopathy compared to the control group.

In a study investigating IMA levels in glaucoma, Karakurt et al. [28] compared the amount of pro-oxidant molecules and IMA levels in 70 open-angle glaucoma patients and found that both values were significantly higher in the patient group. They associated this with oxidative stress. Chang et al. [29] found that IMA levels were significantly higher in patients with primary angle-closure glaucoma in their study evaluating oxidative stress parameters.

In a study evaluating the relationship between IMA levels and oxidant/antioxidant profile in cataract patients [30], IMA and MDA levels were significantly higher in the cataract group than in the control group, while SOD and CAT levels were lower. In this study, high IMA and MDA levels were associated with oxidative stress. In our study, serum IMA levels were significantly higher in the KC group than in the control group. Consistent with other studies, we hypothesize that elevated IMA levels in patients with KC are attributable to oxidative stress.

MDA is a product of ROS-induced lipid peroxidation. Therefore, MDA levels are indicators of lipid peroxidation and oxidative stress [14]. In their study, Kılıc et al. [8] compared serum MDA levels of the KC group and the control group and found MDA levels significantly higher in the KC group, similar to our study. In our study, serum MDA levels were significantly higher in the KC group than in the control group (*p* < 0.001). Buddi et al. [5] compared MDA levels in 26 KC and 11 normal corneas and found MDA levels significantly higher in the patients with KC.

Total thiol (TT) levels reflect the capacity of systemic antioxidant defenses and are essential for maintaining redox homeostasis by neutralizing reactive oxygen species (ROS). Thiol groups (–SH), which are predominantly present in plasma proteins and low-molecular-weight antioxidants such as glutathione, are rapidly oxidized under oxidative stress, leading to disulfide bond formation and reduced TT concentrations. Consequently, decreased serum TT levels are considered a reliable indicator of increased oxidative burden and weakened antioxidant defense in various pathological conditions, including ocular diseases associated with oxidative damage [5,14].

In the present study, serum TT levels were significantly lower in patients with keratoconus compared to healthy controls, suggesting a disruption of antioxidant defense mechanisms in keratoconus. This decrease may be attributed to increased utilization of thiol-containing antioxidants in response to excessive ROS production in keratoconic corneal tissue. Reduced TT levels may contribute to oxidative modification of structural proteins, extracellular matrix degradation, and stromal thinning, which are characteristic features of keratoconus. Consistent with previous studies demonstrating elevated oxidative stress and reduced antioxidant capacity in KC, our findings support the role of impaired thiol-mediated antioxidant defense in disease pathogenesis and highlight TT as a potential biomarker and therapeutic target for KC management [5,8,14].

Although keratoconus is primarily a localized corneal disorder, systemic oxidative stress markers may reflect the overall redox imbalance contributing to corneal pathology. Therefore, serum biomarkers should be interpreted as indirect indicators rather than direct measures of corneal oxidative status.

The lack of a significant association between oxidative stress markers and KC stage may be partly attributable to the limited sample size, particularly in advanced stages, resulting in reduced statistical power.

From a clinical perspective, decreased serum total thiol levels may provide additional insight into systemic oxidative stress in patients with KC. Measurement of TT, together with established oxidative stress markers such as IMA and MDA, may contribute to a more comprehensive understanding of the pathophysiology of KC. While these findings provide supportive evidence for a potential role of oxidative stress, they should be interpreted with appropriate caution and do not imply immediate diagnostic or therapeutic applicability. Instead, these biomarkers may be considered as promising candidates for further investigation, particularly in clarifying their potential contribution to future preventive or adjunctive therapeutic strategies.

These findings may help to better characterize the systemic oxidative profile of KC patients and could contribute to risk stratification or disease monitoring in future studies.

## 5. Conclusions

In conclusion, reduced serum total thiol levels in patients with KC indicate impaired antioxidant defense mechanisms associated with increased oxidative stress. Along with elevated IMA and MDA levels, TT may serve as a complementary biomarker for assessing oxidative imbalance in KC. These findings suggest a potential association between oxidative stress and KC; however, they should be considered exploratory and hypothesis-generating. Further large-scale, longitudinal, and mechanistic studies are required to determine the clinical utility of these biomarkers.

## 6. Limitations of the Study

The main limitations of this study include the relatively small sample size and its single-center, cross-sectional design, which may limit the generalizability of the findings and preclude causal inferences. The small number of patients in advanced KC stages may have limited the ability to detect stage-related differences, and larger studies are needed to clarify this relationship. Although subgroup analysis was performed by combining advanced stages, the relatively small number of patients in severe KC groups may still limit the ability to detect stage-dependent differences.

Although KC is primarily a localized corneal disorder, the use of systemic oxidative stress markers may be considered a relative limitation of this study. Serum biomarkers are more likely to reflect the overall redox status of the body rather than the specific oxidative microenvironment of the cornea. However, they may still provide valuable supportive information regarding the systemic component of oxidative stress. Therefore, these findings should be interpreted as indirect but complementary indicators of the oxidative processes potentially involved in the pathophysiology of KC. In addition, oxidative stress markers were measured only in serum and may not fully reflect local oxidative processes in the cornea in KC. Despite these limitations, the consistent alterations observed in IMA, MDA, and TT levels support the relevance of oxidative stress in KC and provide a basis for future longitudinal and multicenter studies.

Another limitation is the lack of aqueous humor or tear fluid measurements, which could provide a more direct assessment of local oxidative stress.

## Figures and Tables

**Table 1:** Mean serum ischemia-modified albumin, malondialdehyde, and total thiol values in the keratoconus and control groups.

	KC Group (*n* = 40) Mean ± SD	Control Group (*n* = 43) Mean ± SD	*p*-Value
**IMA (Abs.U)**	2.52 ± 0.07	1.2 ± 0.03	**˂** **0.001**
**MDA (mmol/L)**	1.68 ± 0.05	0.74 ± 0.04	**˂** **0.001**
**TT (mmol/L)**	0.82 ± 0.41	2.23 ± 0.04	**˂** **0.001**

KC (keratoconus); IMA (ischemia-modified albumin); MDA (malondialdehyde); TT (total thiol).

## Data Availability

Data and materials are available.
